# Xanthohumol Sensitizes Melanoma Cells to Vemurafenib by Lowering Membrane Cholesterol and Increasing Membrane Fluidity

**DOI:** 10.3390/ijms26052290

**Published:** 2025-03-04

**Authors:** Marine Devinat, Jessica Thevenard-Devy, Fatiha Ghilane, Jerome Devy, Lise Chazee, Christine Terryn, Laurent Duca, Emmanuelle Devarenne-Charpentier, Hassan El Btaouri

**Affiliations:** 1 UMR-CNRS 7369 Matrice Extracellulaire et Dynamique Cellulaire (MEDyC), UFR Sciences Exactes et Naturelles, Université de Reims Champagne Ardenne, Moulin de la Housse, BP 1039, 51687 Reims, CEDEX, France; marine.devinat@univ-nantes.fr (M.D.); jessica.devy@univ-reims.fr (J.T.-D.); jerome.devy@univ-reims.fr (J.D.); lise.chazee@univ-reims.fr (L.C.); laurent.duca@univ-reims.fr (L.D.); emmanuelle.charpentier@univ-reims.fr (E.D.-C.); 2 Laboratoire de Biologie des Pathologies Humaines, Université Mohammed V de Rabat, 4 Avenue Ibn Battouta, Rabat B.P. 1014 RP, Morocco; fatiha.ghilane@um5r.ac.ma; 3 Plateau Technique en Imagerie Cellulaire et Tissulaire (PICT) Pôle Santé, UFR Pharmacie, Université de Reims Champagne Ardenne, 51 Rue Cognacq Jay, 51096 Reims, France; christine.terryn@univ-reims.fr

**Keywords:** natural compound, chemoresistance, melanoma cancer, membrane fluidity, xanthohumol, vemurafenib

## Abstract

Chemoresistance remains one of the major obstacles to cancer treatment. The search for specific molecules that could improve cancer treatment has become one of the objectives of biomedical research. Identifying new natural molecules to enhance chemotherapy treatment or improve sensitization to conventional therapies has become a key objective. Here, we evaluated the effect of Xanthohumol (XN) extracted from hop on SKMEL-28 melanoma cells and their sensitization to vemurafenib (VEM) treatment. We measured the XN effect on cell viability and apoptosis. We also assessed the effect of XN on membrane fluidity and membrane cholesterol levels. Finally, we studied the impact of XN on cell sensitization to VEM. Here, we showed that XN reduced SKMEL-28 cell viability through an apoptotic mechanism. Our results demonstrated the potential role of XN in sensitizing cancer cells to VEM with a less toxic effect on non-tumor cells. A study of XN’s molecular mechanism showed that XN was able to induce cholesterol depletion and increased fluidity in SKMEL-28 cancer cells. This leads to an increase in VEM incorporation. Here, we describe the importance of the strategy to modulate membrane fluidity by XN in order to significantly improve anticancer therapy.

## 1. Introduction

Biological membranes are complex arrangements principally composed of phospholipids and both integral and peripheric proteins [1]. The primary function of biological membranes is to act as a selective permeability barrier separating the extracellular environment from the intracellular medium [2]. It now seems clear that the biological membrane modulates a multitude of cellular functions such as transport, respiration, and transmission of biological information. Homeostatic regulation of key membrane parameters, such as lipid composition or fluidity, is vital for cell function. It is also involved in tumor cell invasion, migration, cell death pathways, and metastasis processes [3,4,5,6,7,8,9]. Both the modulation of the composition and physical properties of membrane lipids change their fluidity. Depending on the temperature, lipids change from a solid-ordered gel phase (So) to a disordered liquid state (Ld). At physiological temperature, most membranes are in the Ld state due to their lipid composition [10,11,12].

Changes occurring in membrane fluidity play a key role in the regulation of membrane properties under physiological conditions and in the pathogenesis of disease [13,14,15,16,17]. In recent decades, the role of the plasma membrane, especially its biophysical state in a tumor context, is increasingly recognized in the field of anticancer therapies. Genetic alterations, responsible for changes in the lipid profile and biophysical properties of the membrane, affect cancer cell motility and the potential of metastatic cells [18,19,20,21]. It also reduces the free diffusion of anticancer drugs across the plasma membrane. As a consequence, tumor cell sensitivity to chemotherapy is decreased, leading to the development of chemoresistance [22,23]. This resistance can be clinically acquired and established after a positive response to treatment, or intrinsic and present in the absence of any chemotherapeutic treatment [24,25].

Membrane fluidity is also strongly influenced by membrane domains characterized by composition different from the rest of the membrane [26,27]. These cholesterol-enriched domains serve as platforms for the distribution of membrane proteins [28,29]. Cholesterol is an essential component of cell membranes, necessary for the organization of membrane lipids. It plays an integral role in cell structure and function as well as in the regulation of cellular processes such as metabolism, compartmentalization, environmental regulation, and both extracellular and intracellular communication [28,29]. Alterations in cholesterol distribution have been demonstrated in neurodegenerative diseases [29], tumor metastases [14].

Recently, plasma membranes appeared as a possible target for the development of new anticancer strategies. Indeed, most cancer cells modify their membrane lipid composition to promote not only proliferation and/or resistance to therapies but also to alter the pharmacokinetic and pharmacodynamic parameters of anticancer drugs [23,30,31,32,33,34]. Different classes of drugs have therefore been evaluated with the aim of modulating the biochemical and biophysical characteristics of cancer cell membranes [22]. This would undoubtedly enhance their role as new therapies or adjuvants to existing therapies.

Several natural products and their secondary metabolites have revealed immense pharmacological and biological properties. Indeed, studies have shown that terpenes can reduce the incidence of tumor progression. Other studies have concluded that flavonoids and stilbenes were able to sensitize tumor cells to chemotherapeutic treatments and induce apoptosis [35,36,37,38,39,40].

Although the molecular mechanisms of action of some of these products are not yet elucidated, extensive research in this field continues to generate new data that can be used in the clinic [41,42,43,44,45]. Some recent studies have suggested that these natural products may interact with membranes and that these interactions may be the origin of their beneficial effects both in cancer prevention and cancer therapy [46,47,48,49,50].

Xanthohumol (3′-[3,3-dimethyl allyl]-2′,4′,4-trihydroxy-6′-methoxychalcone), the natural flavonoid mainly found in hop (*Humulus lupulus* L.), is suggested to have anticancer properties that inhibit the proliferation and induction of human cancer cell apoptosis [51,52,53,54,55,56,57]. Interest in XN activities has grown steadily since the 1990s, with scientific research initiated worldwide, and an increasing number of publications and patents on the subject [58]. Despite its pharmacological activity, it is not known whether xanthohumol has chemo- and radio-sensitizing activities on drug-resistant cancer cells.

In our work, we focused on the role of Xanthohumol (XN) in the modulation of human melanoma SK-MEL-28 cell membrane fluidity and its impact on the sensitization of these cells to VEM treatment. It has been shown that melanoma development is related to the phosphorylation-induced activation of two signaling pathways: PI3K/PTEN/AKT and MAPK (RAS/RAF/MEK/ERK) [59]. The BRAFV600E mutation occurs very frequently (more than 60%) in melanoma [60]. BRAF is therefore the main target for drug research and development [61,62]. VEM, BRAF expression inhibitor, has been tested for the treatment of melanoma both in vitro and in vivo and has yielded positive results [63]. However, an in vitro study showed recurrence and chemoresistance related to pERK reactivation [64]. Numerous combination treatments of VEM and naturally occurring products in vitro have reduced the cancerous properties of melanoma cells [65,66].

## 2. Results

**Figure 1:**
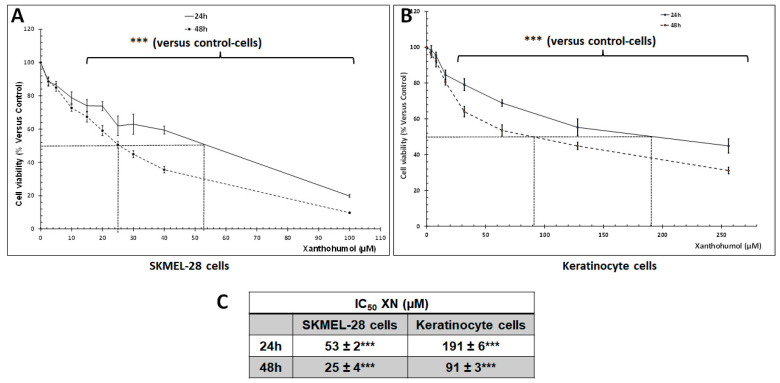
Cytotoxic effect of XN on the SKMEL-28 cells and keratinocyte cells. (**A**,**B**) SKMEL-28 cells and Keratinocyte cells were incubated with XN at different concentrations. After 24 and 48 h, cell viability was measured by UptiBlue kit Assay using Infinite 200 PRO plate reader (TECAN, Männedorf Switzerland). The results were calculated as a percentage of control and represented with standard deviation (S.D.) of at least three independent experiments. The statistical significance of differences was calculated using the Student’s test. *** *p* < 0.001 compared to control cells. (**C**) Summary table of IC_50_. IC_50_ was the concentration of XN required for 50% inhibition of cell viability. It was obtained by comparing treated and untreated cells.

We evaluated the cytotoxic effect of XN on the SKMEL-28 cells. To that end, cells were treated with various XN concentrations (0–100 µM) for 24 and 48 h, and cell viability was measured by spectrofluorometry. As shown in Figure 1A,C, XN decreased SKMEL-28 cell viability in a dose-dependent manner with an IC_50_ = 53 and 25 μM for a period of 24 and 48 h, respectively. We also tested the XN effect on non-tumoral keratinocytes cells (Figure 1B,C). The cells were treated with XN at various concentrations (0–250 µM) for 24 and 48 h and cell viability was measured by spectrofluorometry. The results showed that XN has a 3-fold higher IC_50_ for non-tumor cells than for SKMEL-28 cells with an IC_50_ = 191 and 91 μM for a period of 24 and 48 h, respectively. This suggested that tumor cells were more sensitive to XN treatment than healthy cells.

**Figure 2:**
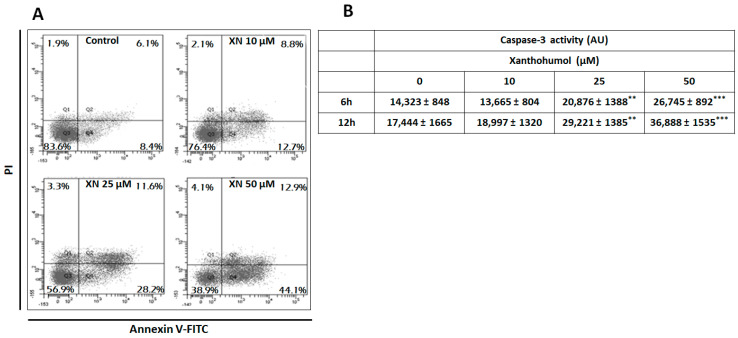
XN−induced caspase−3 activation and apoptosis in SKMEL−28 cells. SKMEL-28 cells were incubated with XN at three concentrations. (**A**) After 24 h, apoptosis was measured by Annexin V−FITC/PI Apoptosis Staining Detection. The percentage of apoptotic cells was calculated from values obtained by flow cytometry. (**B**) Activity was measured by a caspACE assay kit. The results obtained from three independent experiments were represented with standard deviation (S.D.). Student’s *t*−test was used for the statistical significance of different values. ** *p* < 0.01 and *** *p*< 0.001 compared to control.

To determine if XN inhibited cell viability by inducing apoptosis, cells were incubated with XN at three concentrations (10, 20, and 50 µM) for 24 h, and apoptosis was determined by flow cytometry. Figure 2A showed that XN induced apoptosis in SKMEL-28 cells, and the apoptosis rate increased in a dose-dependent manner to 12.7, 28.8, and 44.1%, respectively, for 10, 25, and 50 µM of XN compared to untreated cells. To confirm the pro-apoptotic effect of XN, we analyzed the activity of the caspase-3 (Figure 2B). We showed that XN induced the cleavage of pro-caspase-3 in correlation with an increase in the caspase-3 activity. These results confirmed that XN reduced cell viability via caspase-3 activation in SKMEL-28 cells.

**Figure 3:**
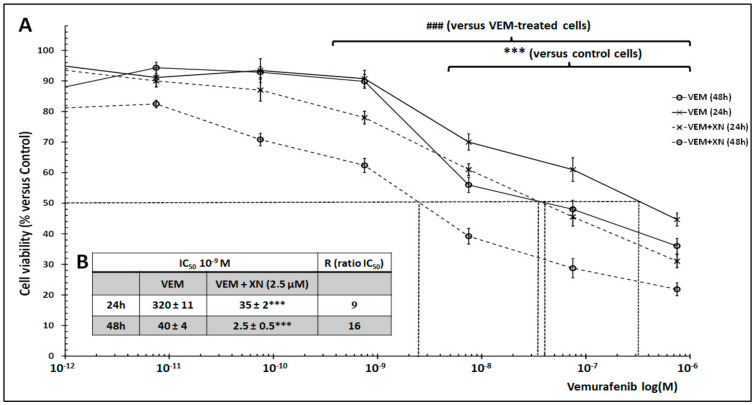
XN−induced SKMEL-28 cell sensitization to Vemurafenib. (**A**) SKMEL-28 cells were incubated with VEM at concentrations ranging from 10^−11^ to 10^−6^ M with or without 2.5 µM XN. After 24 and 48 h, cell viability was measured using UptiBlue Viable Cell Counting Assay. The results were calculated as a percentage of control and represented with standard deviation (S.D.) of at least three independent experiments. The statistical significance of differences was calculated using the Student’s test. *** *p* < 0.001 compared to control cells and ### *p* < 0.001 compared to VEM treated cells. (**B**) Summary table of IC_50_ and R (IC_50_ cell cultured with XN/IC_50_ cell cultured without XN) in each treatment condition.

Considering the cytotoxic effect of XN, we thus hypothesized that it could sensitize SKMEL-28 cells to VEM. Therefore, cells were treated with different concentrations of VEM for 24 and 48 h with or without sublethal dose XN (2.5 µM), and cell viability was measured by spectrofluorometry (Figure 3). VEM alone reduced SKMEL-28 cell viability with an IC_50_ = 320 × 10^−9^ and 35 × 10^−9^ M for 24 and 48 h, respectively. Interestingly, XN sensitized SKMEL-28 cells to VEM treatment. Indeed, IC_50_ was reduced by 9-fold and 16-fold for 24 and 48 h, respectively, as compared to VEM-treated cells.

**Figure 4:**
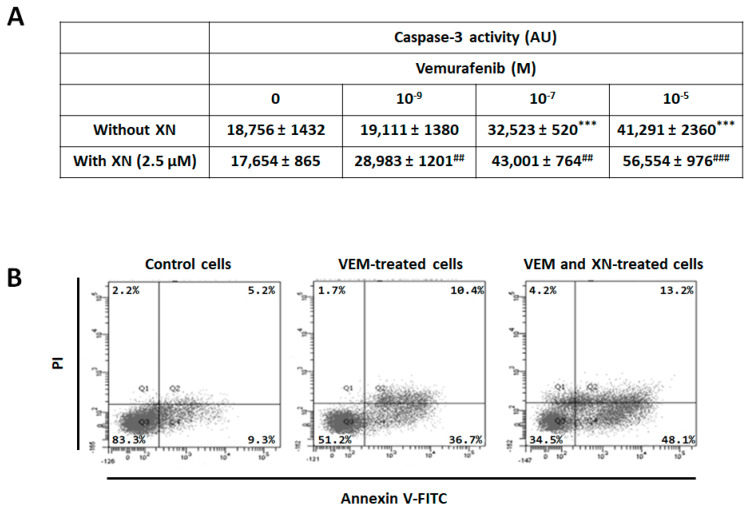
XN potentiated the VEM’s apoptotic effect on SKMEL-28 cells. (**A**) SKMEL-28 cells were incubated with VEM at three concentrations (10^−9^, 10^−7^, and 10^−5^ M) with or without 2.5 µM XN. After 12 h, caspase-3 activity was measured by a caspACE assay kit. The results obtained from three independent experiments were represented with standard deviation (S.D.). Student’s *t*-test was used for the statistical significance of different values. *** *p* < 0.001 compared to control cells. ## *p* < 0.01 and ### *p* < 0.001 compared to VEM treated cells. (**B**) SKMEL-28 cells were incubated with VEM (10^−6^ M) with or without 2.5 µM XN. After 24 h, apoptosis was measured by Annexin V-FITC/PI Apoptosis Staining Detection. The percentage of apoptotic cells was calculated from values obtained by flow cytometry.

This drug sensitizer effect of XN was confirmed by apoptosis and caspase-3 activity analysis. Indeed, Figure 4 showed that XN potentiated the VEM-induced apoptosis of SKMEL-28 cells. This effect was confirmed by the activity of caspase-3 which is amplified following XN treatment SKMEL-28 cells.

**Figure 5:**
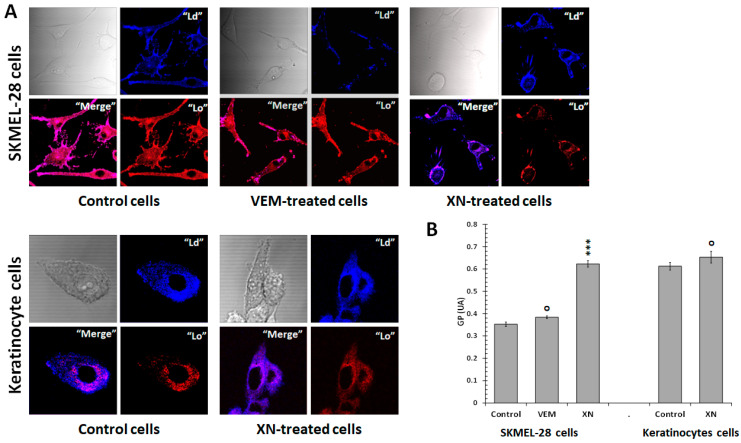
XN-increased SKMEL-28 cell membrane fluidity without affecting keratinocyte cells. (**A**) SKMEL-28 cells and keratinocytes cells were incubated with 10^−7^ M VEM or 2.5 µM XN. After 12 h, the cells were suspended in Laurdan solution and incubated at 37 °C for 15 min. The fluorescence was measured by Confocal microscopy as indicated in the Section 4. (**B**) GP was calculated according to where IB 435 and IR 500 refer to the average (I_B_ − I_R_)/(I_B_ − I_R_). The results obtained from three independent experiments were represented with standard deviation (S.D.). The Student’s *t*-test was used for the statistical significance of different values. *** *p* < 0.001 and **^O^** NS compared to control cells.

We, therefore, explored whether the XN sensitizer effect could implicate modulation of the SKMEL-28 membrane fluidity cells. To that end, the phospholipid organization was investigated by visualizing the fluorescence distribution of Laurdan in the SKMEL-28 cell membrane. Laurdan is a fluorescent dye used to measure variation in membrane fluidity. Indeed, the Laurdan emission spectrum varies from blue in the disordered lipid phase of the membrane “Ld” to red in the ordered lipid phase “Lo”. Thus, SKMEL-28 cells were treated with XN (2.5 µM) or VEM (10^−9^ M) for 12 h and incubated with Laurdan (5 µM) for 15 min and then analyzed by confocal microscopy. We also tested the XN effect on non-tumoral keratinocytes cells (Figure 5). Ld/Lo phases were quantified by calculating the generalized polarization (GP). In untreated cells, the GP value was lower in SKMEL-28 cells than in non-tumor Keratinocytes cells. This proved that in SKMEL-28 cells, the ordered phase (Lo) was dominant, suggesting a lower membrane fluidity in SKMEL-28 cells compared to keratinocyte cells. VEM treatment did not modify the GP value. Treatment of the cells with XN induced an increase in the GP value from 0.34 to 0.6, characterized by a higher disordered phase (Ld). This result suggested that XN increased membrane fluidity in SKMEL-28 cells. However, keratinocyte cells were not affected by XN treatment. In fact, keratinocyte cells exhibited a fluid membrane structure in the absence of treatment.

**Figure 6:**
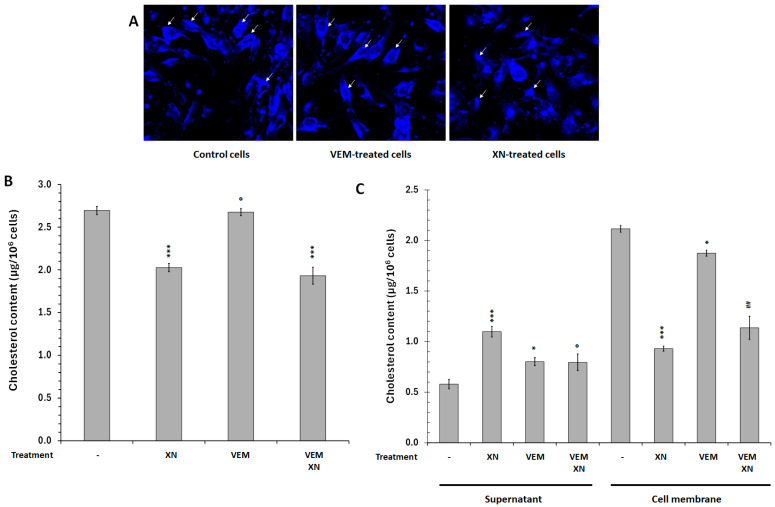
XN-modulated SKMEL-28 cell subcellular cholesterol distribution. SKMEL-28 cells and keratinocytes cells were incubated for 12 h with 10^−7^ M VEM or 2.5 µM XN. (**A**) Cellular distribution of cholesterol was visualized using filipin labeling. The fluorescence was measured by Confocal microscopy as indicated in the Section 4. The results were obtained from three independent experiments. Arrows indicated accumulation of membrane cholesterol. (**B**,**C**) After cytosol and cell membrane isolation, cholesterol levels were measured in the 2 cell fractions using Cholesterol Assay Kit (Cell-Based). The results obtained from three independent experiments were represented with standard deviation (S.D.). The Student’s *t*-test was used for the statistical significance of different values. * *p* < 0.1 *** *p* < 0.001 and **^O^** NS compared to control cells, ## *p* < 0.01 compared to VEM treated cells.

Cell membranes contain a variety of lipid species that differ in their physicochemical properties and exhibit membrane heterogeneities. Cholesterol is the major sterol in animal cell membranes, representing on average 30% of the lipid bilayer. Cholesterol plays a crucial role in maintaining structural integrity and regulating the fluidity of cell membranes [67,68,69,70]. We, therefore, examined the cellular distribution of cholesterol in VEM- or XN-treated cells using filipin labeling, a naturally fluorescent polyene antibiotic that binds to cholesterol, and an enzyme assay kit. SKMEL-28 cells were treated with XN (2.5 µM) or VEM (10^−9^ M) for 12 h and filipin fluorescence was observed by confocal microscopy. Figure 6A showed that in untreated SKMEL-28 cells, filipin labeling, although widely distributed, was accumulated on the cell surface. Treatment of the cells with XN induced an alteration in cholesterol distribution, with the appearance of less intense labeling at the membrane level. However, VEM did not significantly affect filipin labeling. These results confirmed that XN altered the cellular distribution of cholesterol with a decrease in the membrane area. This result was correlated with an increase in GP value. To confirm this hypothesis, cholesterol level was measured in cytosolic and membrane cell fractions Figure 6B showed that XN slightly reduced the amount of total cellular cholesterol, while VEM had no effect. We also showed that cholesterol is mainly found in plasma membrane. It represented 77% of total cellular cholesterol. These results concurred with previous work showing that cholesterol levels were highest at the mammalian cell plasma membrane [71,72]. In the presence of XN, we observed a reduction in membrane cholesterol levels, in parallel with its cytosolic increase (Figure 6C). This result confirmed that XN induced cholesterol depletion in SKMEL-28 cells. VEM had no significant effect on cellular cholesterol distribution.

**Figure 7:**
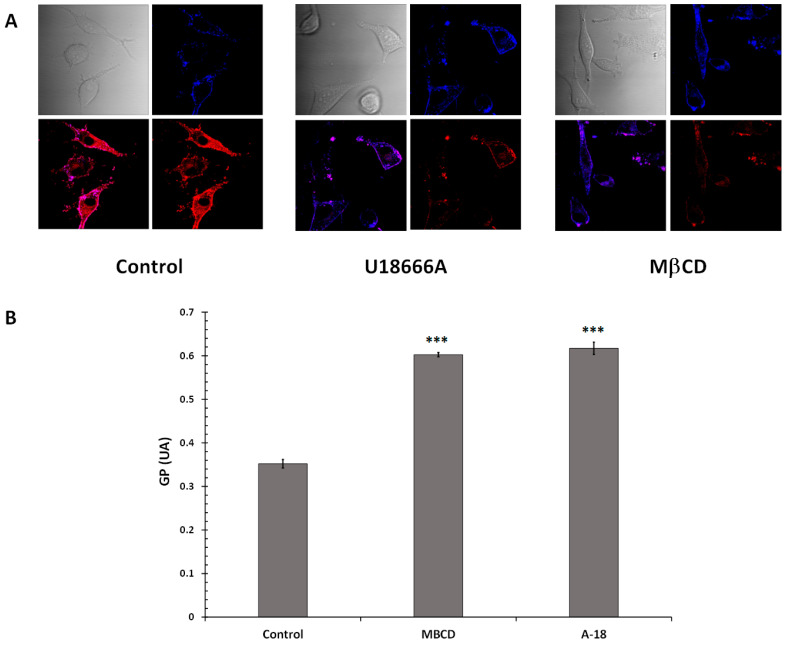
U18666A and MβCD-increased SKMEL-28 cell membrane fluidity. (**A**) SKMEL-28 cells were incubated with MβCD (10 µM) or U18666A (1.5 µM) for 12 h and were suspended in Laurdan solution and incubated at 37 °C for 15 min. The fluorescence was measured by Confocal microscopy as indicated in the Section 4. (**B**) GP was calculated according to where IB 435 and IR 500 refer to the average (I_B_ − I_R_)/(I_B_ − I_R_). The results obtained from three independent experiments were represented with standard deviation (S.D.). The Student’s *t*-test was used for the statistical significance of different values. *** *p* < 0.001 compared to control cells.

Cholesterol depletion is widely used to study the effect of cholesterol on the plasma membrane. It can be achieved through the use of methyl-β-cyclodextrin (MβCD) and the amphiphilic amino-steroid (U18666A) [73,74]. Thus, we used these two inhibitors to confirm a link between cholesterol depletion and increased membrane fluidity in SKMEl-28 cells. To that end, the cells were treated with MβCD (10 µM) or U18666A (1.5 µM) for 12 h and incubated with Laurdan (5 µM) for 15 min at 37 °C (Figure 7). The result showed that MβCD and U18666A induced an increase in the disordered phase (Ld) compared to control cells. It was then confirmed by the quantification of the GP value which increased from 0.34 to 0.6. Indeed, MβCD and U18666A increased GP value. These results suggested that cholesterol depletion could increase SKMEL-28 cell membrane fluidity at physiological temperatures.

**Figure 8:**
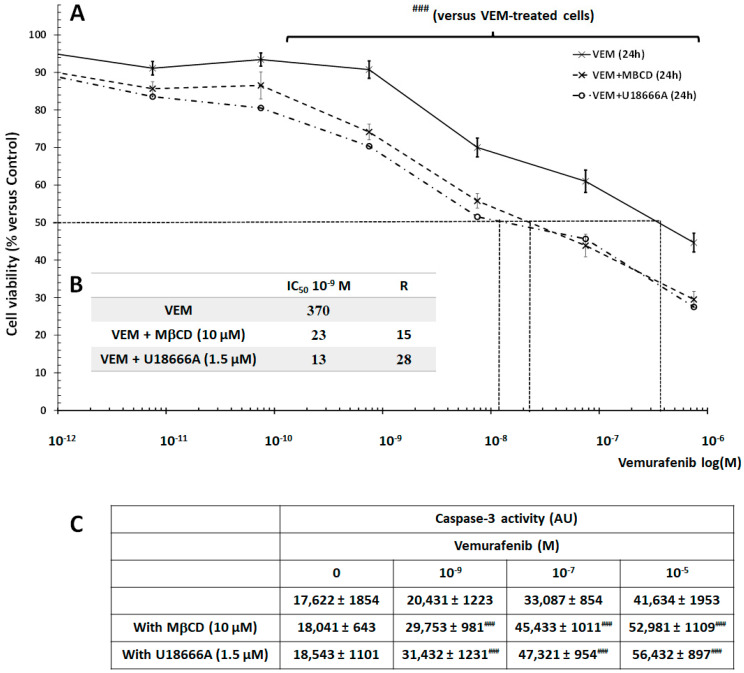
U18666A and MβCD-induced SKMEL-28 cell sensitization to Vemurafenib. (**A**) SKMEL-28 cells were incubated with VEM at concentrations ranging from 10 to 11 to 10-6 M with or without MβCD (10 µM) or U18666A (1.5 µM). After 24, cell viability was measured using UptiBlue Viable Cell Counting Assay. The results were calculated as a percentage of control and represented with standard deviation (S.D.) of at least three independent experiments. The statistical significance of differences was calculated using the Student’s test. ### *p* < 0.001 compared to VEM-treated cells. (**B**) Summary table of IC50 and R (IC50 cell treated by VEM with U18666A or MβCD/IC50 cell cultured with only VEM. (**C**), SKMEL-28 cells were incubated with VEM at three concentrations (10-9, 10-7, and 10-5 M) with or without MβCD (10 µM) or U18666A (1.5 µM). After 12 h, caspase-3 activity was measured by a caspACE assay kit. The results obtained from three independent experiments were represented with standard deviation (S.D.). Student’s *t*-test was used for the statistical significance of different values. ### *p* < 0.001 compared to VEM-treated cells.

To complete our study, we investigated the SKMEL-28 cell sensitization to VEM following treatment by MβCD or U18666A. The cells were treated with different concentrations of VEM for 24 h with or without MβCD (10 µM) or U18666A (1.5 µM) and cell viability was measured by spectrofluorometry. As shown in Figure 8A, MβCD and U18666A sensitized SKMEL-28 cells to VEM treatment and reduced IC_50_ by 15-fold and 28-fold for MβCD and U18666A, respectively, compared to VEM-treated cells. These results were confirmed by caspase-3 activation assay (Figure 8B) supporting the hypothesis that cholesterol depletion by XN could modulate membrane fluidity and cell sensitization to VEM treatment.

**Figure 9:**
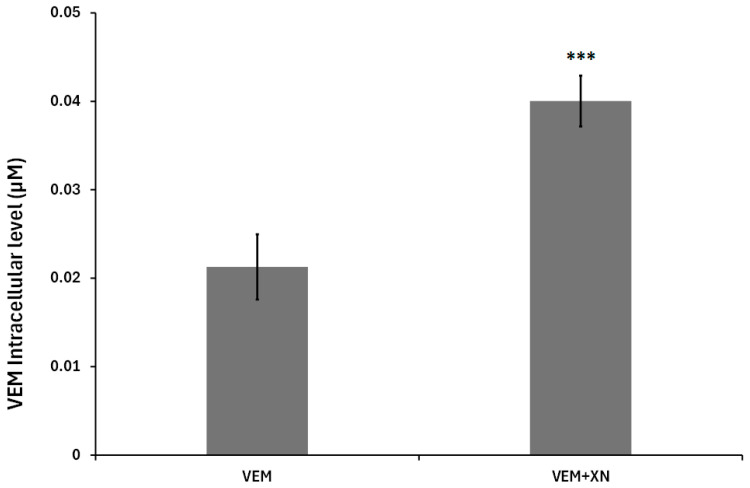
XN-increased vemurafenib incorporation into SKMEL-28 cell. SKMEL-28 cells were incubated with VEM (10^−6^ M) with or without XN (2.5 µM). After 24, VEM incorporated into the cells was measured by HPLC. The results were calculated using a standard range of VEM and represented with standard deviation (S.D.) of at least three independent experiments. The statistical significance of differences was calculated using the Student’s test. *** *p* < 0.001 compared to VEM-treated cells.

To establish a link between the modulation of membrane fluidity and sensitization of SKMEL-28 cells to VEM, we measured the incorporation of this drug following cell treatment with XN. We therefore analyzed by chromatography VEM incorporated level in cells (Figure 9). The results showed that XN treatment induced an increase in VEM incorporation into SKMEL-28 cells. This confirmed the VEM sensitization cells following XN treatment.

## 3. Discussion

Melanoma is an aggressive form of skin cancer characterized by a poor prognosis. Oncogenic mutations in BRAF are common in 50% of melanomas establishing intrinsic resistance linked to constitutive activation of the BRAF/MEK/ERK signaling pathway and leading to uncontrolled cell proliferation [24,60]. In recent decades, new therapies specifically targeting the BRAF oncogene have improved the patients’ prognosis [75]. BRAF inhibitors such as VEM are currently approved as first-line treatments for patients with mutated BRAF metastatic melanoma [67,76,77]. Unfortunately, although a good initial response to VEM, the treatment induces the development of secondary acquired drug resistance in most patients [78]. Several mechanisms are thought to be responsible for this acquired resistance, including secondary mutations, signaling bypass and activation of other compensatory downstream effectors, and changes in the tumor microenvironment [79,80]. Therefore, the increasing of tumoral chemoresistance required the development of therapeutic approaches posing lower cytotoxic effects and resistance. Several natural products and their derivatives possess important pharmacological properties. Some have considerable anticancer potential. Although the molecular mechanisms of action of some of these products have yet to be elucidated, extensive research in this field continues to generate new data for identifying the interactions of natural products and their derivatives with cancer cells, for use in “targeted oncology therapies”. These products preferentially involve multiple mechanistic pathways able to overcome or bypass chemoresistance in several tumor types [41].

In this context, we evaluated the potential therapeutic effect of Xanthohumol (XN) on SKMEL-28 melanoma cells and its capacity to sensitize these cells to VEM conventional treatment. The structure of XN was first identified by Verzele et al. [81]. However, the pharmacological properties of XN were identified in the 1990s, in particular antibacterial, antiviral, antifungal, antioxidant, and anti-inflammatory activities [82]. In recent years, more evidence has suggested the anticancer activity of XN against several cancer [83]. We showed that XN reduced SKMEL-28 cell viability in a dose- and time-dependent manner through a caspase-3-dependent apoptotic mechanism. The IC_50_ values determined for SKMEL-28 cells in this study were at the micromolar range (IC_50_ = 53 and 25 μM for a period of 24 and 48 h, respectively) and in accordance with other values obtained in human colon adenocarcinoma and liver carcinoma cell lines [84].

The biggest problem of chemotherapy has been its inability to distinguish between cancer cells and normal cells, resulting in significant toxicity and secondary effects. Over the past two decades, cancer treatment has undergone a major shift from broad-spectrum cytotoxic drugs to targeted drugs with the aim of sparing non-tumor cells [85,86]. Our experimental results also showed that the IC_50_ of XN on non-tumor cells such as keratinocytes was 3 times higher than KMEL-28 cells. These results confirmed that XN was less toxic to non-tumor cells and suggested that XN induced a selective cytotoxic effect that targeted tumor cells at low doses. XN could be considered a targeted therapy since it was able to induce a cytotoxic effect in SKMEL-28 cells without affecting non-tumoral keratinocyte cells. The selective anticancer effect of natural substances has been confirmed by other studies, which have shown that the anticancer effect of these substances was related to their ability to induce apoptosis in cancer cells without cytotoxic effects on healthy cells [86,87,88].

Cancer treatment with natural compounds has shown promising results against various malignant tumors [88,89]. Several studies have confirmed the use of natural substances as adjuvants in combination with different types of anti-tumor therapies, such as chemotherapy, radiotherapy, and immunotherapy [87,90]. In this context, it has been suggested to study the anti-tumor activity of natural compounds and their synergetic efficacity with traditional anticancer drugs. In our study, we showed that sublethal dose XN enhanced the cytotoxic effect of VEM by reducing the IC_50_ by 9-fold and 16-fold for 24 and 48 h, respectively. These results demonstrated the potential role of XN in sensitizing cells to VEM thus leading to apoptosis. Thus, XN can be employed as an adjuvant to improve the SKMEL cell’s sensitization to VEM treatment.

The molecular mechanism of natural compounds has conventionally involved enzymes, receptors, ion channels, and transporters [91,92]. However, it is crucial for natural compounds to cross the lipid environment of the plasma membrane. Therefore, their interactions with the constituent lipids of the cellular membrane are considered one of the important mechanisms underlying the action of natural compounds. These interactions are thought to affect the physicochemical properties of membranes, including fluidity, microviscosity, orderliness elasticity, and permeability [93]. For example, most flavonoids, like lipophilic compounds, tend to accumulate in biological membranes and thus influence their function by modulating lipid phase behavior [94]. In a tumoral context, alteration of the physical membrane properties can be correlated to a modification of the membrane lipid composition [3,79,95]. Lipidomic studies have shown possible differences in lipid composition and membrane function in tumor cells compared to healthy cells [22]. Indeed, the cholesterol content has been described in multiple studies as a modulator of cancer cell survival and aggressiveness [96]. Based on this, it has been hypothesized that cholesterol lowering in cancer cells could be proposed as a potential anticancer strategy.

In this study, we showed that XN was able to affect the subcellular distribution of cholesterol by decreasing plasma membrane cholesterol levels and increasing cytosolic levels. Cholesterol depletion led to an increased fluidity of SKMEL-28 cancer cells and an increased intracellular concentration of VEM. This resulted in SKMEL-cell sensitization to chemotherapeutic treatment. The involvement of cholesterol in the membrane fluidity was confirmed by using MβCD and U18666A. Indeed, MβCD and U18666A induced an increase in the disordered phase (Ld) and membrane fluidity. Interestingly, we also showed that MβCD or U18666A treatment improved SKMEL-28 cell sensitivity to VEM. These results confirmed the involvement of cholesterol depletion in increasing membrane fluidity and SKMEL-28 cells sensitizing to VEM treatment. Our results were in agreement with Li et al. studies (2006) reporting that cholesterol depletion caused induction of apoptosis in breast epithelial cell lines without affecting normal cells [97]. Indeed, it has been demonstrated that a high cholesterol content in membranes was correlated to their lower fluidity [98,99,100]. This effect was accompanied by an increase in the tumor cell chemoresistance [47,101,102]. XN’s lack of effect on non-tumor keratinocyte cells may be correlated to the membrane of these cells, which has a higher degree of fluidity than that of SKMEL-28 cells.

**Figure 10:**
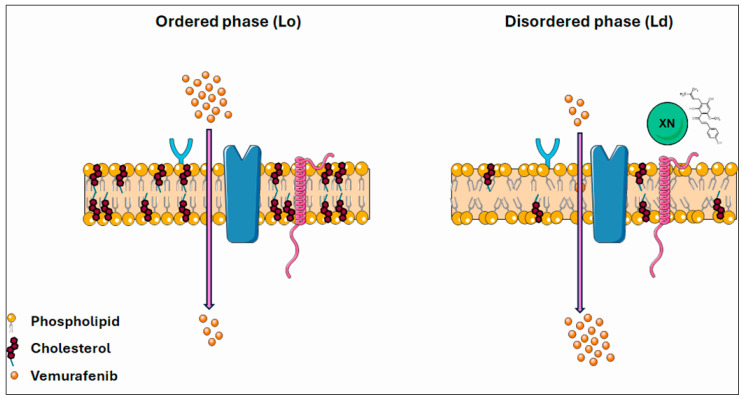
XN enhancement of membrane fluidity and its role in sensitization of SKMEL-28 cells to VEM treatment.

Cholesterol is a major component of animal cell membranes, which is required to maintain integrity and regulate the fluidity of plasma membranes [103,104]. Its effect may be modulated by temperature, but also by the fatty acid composition of membrane phospholipids. In our study, we described the crucial importance of cholesterol in the cell membrane and its involvement in reducing membrane fluidity and supporting SKMEL-28 cell resistance to VEM treatment. Cholesterol depletion by XN increased membrane fluidity, rendering SKMEL-28 cells more sensitive to chemotherapeutic treatment without affecting healthy cells. Thus, strategies to modulate membrane fluidity by XN can significantly improve VEM anticancer treatment in human melanoma cells (Figure 10). Today, it is possible to stabilize XN in a hydrophobic environment by encapsulation in lecithin-based liposomes for antitumor therapeutic applications. Thus, XN could offer a substantial therapeutic value as an adjuvant or an alternative to conventional therapies.

## 4. Materials and Methods

### 4.1. Materials

SK-MEL-28 cells, melanocytes isolated from the skin tissue of a 51-year-old, male patient with malignant melanoma were purchased from ATCC (USA). HaCaT cells, the human immortalized keratinocyte cell line, were purchased from Thermo Fisher Scientific (Dublin, Ireland). Vemurafenib, Xanthohumol, Laurdan, and UptiBlue Viable Cell Counting Kit, were purchased from Interchim (Montluçon, France). Cholesterol/Cholesteryl Ester Assay Kit Quantitation, Cholesterol Assay Kit (Cell-Based), and Caspase-3 Assay Kit (Colorimetric) were from Abcam (Paris, France). FITC Annexin V Apoptosis Detection Kit was purchased from BD Biosciences (Le Pont de Claix, France). Caspase-3, Flotillin-1, and Caveolin-1 antibodies were from Cell Signaling Technology (Saint-Cyr-L’École, France). U18666A, Methyl-βCyclodextrin, and all other products were from Sigma Aldrich (Saint-Quentin, France).

### 4.2. Cell Culture

SKMEL-28 cells and HaCaT cells were suspended in DMEM/F12 medium containing 10% (*v*/*v*) fetal calf serum, streptomycin (100 µg/mL), and penicillin (100 IU/mL) and placed in 75 cm^2^ flasks at 37 °C. Cells were then trypsined and cultured in appropriate well plates for cell viability, flow cytometry, confocal microscopy, Western blot analysis, caspase assay, and HPLC.

### 4.3. Cell Viability

Cells were cultured in 96-well plates at 10^4^ cells/mL for 24 h. The medium was then substituted with serum-free medium with or without different agonists. After 24 h and 48 h incubation, 10% (*v*/*v*) UptiBlue diluted (10× in medium) was added for 3 additional hours. The viability was determined by spectrofluorometry as mentioned in the Interchim-UptiBlue™ protocol (λex: 550 nm; λem: 590 nm). The results were calculated as a percentage of control as follows: % of treated viable cells versus untreated viable cells.

### 4.4. Detection of Apoptosis (Annexin V/PI Staining)

Cells were plated in 6-well plates at 2 × 10^5^ cells/mL^−1^ and incubated with different agonists for 24 h. Apoptosis was measured by Annexin V-FITC Apoptosis Staining Detection Kit. Briefly, after treatment, cells were gently trypsinized and washed with DMEM culture medium and collected by centrifugation. Cell pellet (2 × 10^5^ cells) was suspended in 500 µL of 1X binding buffer. 5 µL of annexin V-FITC and 5 µL of propidium iodide (PI) were added. The homogenate was incubated at room temperature for 5 min in the dark. Annexin V-FITC binding was analyzed by flow cytometry (λex = 488 nm; λem = 350 nm) using a FITC signal detector (usually FL1) as mentioned in BD Biosciences protocol and PI staining by the phycoerythrin emission signal detector (usually FL2).

### 4.5. Caspase-3/7 Activity

Cells were washed with ice-cold PBS and scrapped with ice-cold lysis buffer. Caspase-3/7 activity was measured by incubating 50 μg of cytosolic fraction with a Caspase-Glo^®^ 3/7 colorimetric assay and absorbance was measured at 405 nm following its cleavage. Absorbance was measured with a multichannel plate reader (Metertech, Inc. Σ960, Taipei, Taiwan).

### 4.6. Measurements of Membrane Fluidity

Following incubation in a Petri dish containing DMEM medium, the cells were washed three times with ice-cold PBS suspended in Laurdan solution (5 µM in PBS) and incubated at 37 °C for 15 min. The fluorescence was measured by Confocal microscopy images of cells and analyzed by a Zeiss (Oberkochen, Germany) LSM710 Meta confocal microscope using either a ×63 Plan Apochromat objective at a 132 nm.pixel^−1^ resolution, leading to a slight XY oversampling. The temperature was set to 37 °C. BrightLine single-band bandpass filter in front of each detector: 460/80 nm for the blue channel and 540/50 nm for the red channel. The Zen software program (ZEN 3.1 black edition LS.Ink) was used to acquire images as previously detailed [105]. 

GP was calculated according to $\begin{array}{c} GP=\frac{I_{B}-I_{R}}{I_{B}+I_{R}} \end{array}$ where I_B 435_ and I_R 500_ refer to the average emission intensities at those wavelengths [106].

### 4.7. Cholesterol Content Measurement

Cholesterol content was measured using the Cholesterol/Cholesteryl Ester Assay Kit (Abcam) following the manufacturer’s instructions. Cellular distribution of cholesterol was visualized using the Cholesterol Cell-Based Detection Assay kit (Abcam) following the manufacturer’s instructions. The fluorescence was measured by Confocal microscopy images of cells at 37 °C (λex = 340–380 nm; λem = 385–470 nm) The Zen software program was used to acquire images.

### 4.8. Vemurafenib Content Measurement

Cells plated in Petri dish containing DMEM medium were treated by VEM (10^−6^ M) with or without XN (2.5 µM) for 24 h. Cells were centrifuged (3000× *g* for 5 min, 4 °C) and then washed with ice-cold PBS. Cell pellets were lysed by lysis buffer (20 Mm Tris-HCl, 10 mM KCl, 2 mM MgCl_2_, 1 mM EDTA, 0.5 mM DTT, 0.1% NP-40, pH 7.4) and centrifuged 15,000× *g*, 10 min, 4 °C). Supernatant was collected and filtered using microfiltrator Centricon with a 10 KDa cut-off membrane. The filtrate was analyzed by HPLC. The column used is X-TERRA RP-18 (250 × 4.60 mm, ID 5 μm) at a temperature of 30 °C. The mobile phase is a phosphate buffer (12.5 mM) containing acetonitril (60:40 *v*/*v*) at pH 9.0. The DAD detector is calibrated at a wavelength of 249 nm [107].

### 4.9. Statistics

Each experiment was realized at least in triplicate with three independent cultured cells. Data were presented as mean ± SEM. The statistical calculation was based on the Student’s test. *p* values referring to corresponding control are ** and ¥¥ *p* < 0.01, *** and ¥¥¥ *p* < 0.001, **** and ¥¥¥¥ *p* < 0.0001.

## Data Availability

The raw data supporting the conclusions of this article will be made available by the authors upon request.
